# A comparison of Cohen’s Kappa and Gwet’s AC1 when calculating inter-rater reliability coefficients: a study conducted with personality disorder samples

**DOI:** 10.1186/1471-2288-13-61

**Published:** 2013-04-29

**Authors:** Nahathai Wongpakaran, Tinakon Wongpakaran, Danny Wedding, Kilem L Gwet

**Affiliations:** 1 Department of Psychiatry, Faculty of Medicine, Chiang Mai University, Chiang Mai 50200, Thailand; 2 California School of Professional Psychology, Alliant International University, San Francisco, California, USA; 3 Statistical Consultant Advanced Analytics, LLC PO Box 2696, Gaithersburg, Maryland, USA

**Keywords:** Inter-rater reliability, Coefficients, Cohen’s Kappa, Gwet’s AC1, Personality disorders

## Abstract

**Background:**

Rater agreement is important in clinical research, and Cohen’s Kappa is a widely used method for assessing inter-rater reliability; however, there are well documented statistical problems associated with the measure. In order to assess its utility, we evaluated it against Gwet’s AC1 and compared the results.

**Methods:**

This study was carried out across 67 patients (56% males) aged 18 to 67, with a mean SD of 44.13 ± 12.68 years. Nine raters (7 psychiatrists, a psychiatry resident and a social worker) participated as interviewers, either for the first or the second interviews, which were held 4 to 6 weeks apart. The interviews were held in order to establish a personality disorder (PD) diagnosis using DSM-IV criteria. Cohen’s Kappa and Gwet’s AC1 were used and the level of agreement between raters was assessed in terms of a simple categorical diagnosis (i.e., the presence or absence of a disorder). Data were also compared with a previous analysis in order to evaluate the effects of trait prevalence.

**Results:**

Gwet’s AC1 was shown to have higher inter-rater reliability coefficients for all the PD criteria, ranging from .752 to 1.000, whereas Cohen’s Kappa ranged from 0 to 1.00. Cohen’s Kappa values were high and close to the percentage of agreement when the prevalence was high, whereas Gwet’s AC1 values appeared not to change much with a change in prevalence, but remained close to the percentage of agreement. For example a Schizoid sample revealed a mean Cohen’s Kappa of .726 and a Gwet’s AC1of .853 , which fell within the different level of agreement according to criteria developed by Landis and Koch, and Altman and Fleiss.

**Conclusions:**

Based on the different formulae used to calculate the level of chance-corrected agreement, Gwet’s AC1 was shown to provide a more stable inter-rater reliability coefficient than Cohen’s Kappa. It was also found to be less affected by prevalence and marginal probability than that of Cohen’s Kappa, and therefore should be considered for use with inter-rater reliability analysis.

## Background

Clinicians routinely use structured clinical interviews when diagnosing personality disorders (PDs); however, it is common to use multiple raters when researching clinical conditions such as PDs. Because multiple raters are used, it is particularly important to have a way to document adequate levels of agreement between raters in such studies.

The Structured Clinical Interview, based on the Diagnostic and Statistical Manual of Mental Disorders-IV - for Axis II Personality Disorders (SCID II) [1], is one of the standard tools used to diagnose personality disorders. Because this assessment results in dichotomous outcomes, Cohen’s Kappa [2,3] is commonly used to assess the reliability of raters. Only a few studies have assessed inter-rater reliability using SCID II, but our recent report [4] revealed that the overall Kappa for the Thai version of SCID II is .80, ranging from .70 for Depressive Personality Disorder to .90 for Obsessive-compulsive Personality Disorder. However, some investigators have expressed concerns about the low Kappa values found for some criteria, despite the high percentage of agreement [4-6]. This problem has been referred to as the “Kappa paradox” by Feinstein and Cicchetti [7], who stated, “in one paradox, a high value of the observed agreement (P_o_) can be drastically lowered by a substantial imbalance in the table's marginal totals either vertically or horizontally. In the second paradox, kappa will be higher with an asymmetrical rather than symmetrical imbalance in marginal totals, and with imperfect rather than perfect symmetry in the imbalance. An adjusted kappa does not repair either problem, and seems to make the second one worse.” Di Eugenio and Glass [8] stated that κ is affected by the skewed distributions of categories (the prevalence problem) and by the degree to which coders disagree (the bias problem).

In an attempt to fix these problems, Gwet [9] proposed two new agreement coefficients. The first coefficient can be used with any number of raters but requires a simple categorical rating system, while the second coefficient, though it can also be used with any number of raters, is more appropriate when an ordered categorical rating system is used. The first agreement coefficient is called the “first-order agreement coefficient,” or the AC1 statistic, which adjusts the overall probability based on the chance that raters may agree on a rating, despite the fact that one or all of them may have given a random value. A random rating occurs when a rater is not certain about how to classify an object, which can occur when the object’s characteristics do not match the rating instructions. Chance agreement can inflate the overall agreement probability, but should not contribute to the measure of any actual agreement between raters. Therefore, as is done with the Kappa statistic, Gwet adjusted for chance agreement by using the AC1 tool, such that the AC1 between two or multiple raters is defined as the conditional probability that two randomly selected raters will agree, given that no agreement will occur by chance [9]. Gwet found that Kappa gives a slightly higher value than other coefficients when there is a high level of agreement; however, in the paradoxical situation in which Kappa is low despite a high level of agreement, Gwet proposed using AC1 as a “paradox-resistant” alternative to the unstable Kappa coefficient.

Gwet has also proved the validity of the multiple-rater version of the AC1 and the Fleiss’ Kappa statistics, using a Monte-Carlo simulation approach with various estimators [10].

To the best of our knowledge, Gwet’s AC1 has never been tested with an inter-rater reliability analysis of personality disorders; therefore, in this study we analyzed the data using both Cohen’s Kappa and Gwet’s AC1 to compare their levels of reliability.

## Methods

This project was approved by the Ethics Committee of the Faculty of Medicine, Chiang Mai University.

### Subjects

A total of 67 subjects were recruited from the inpatient and outpatient departments of Maharaj Nakorn Chiang Mai Hospital, part of the Faculty of Medicine at Chiang Mai University. Slightly over half (55%) of the subjects were female, and the mean age was 44.07 ± 13.09 years (18 to 67). With regard to the Axis I diagnoses, 30% had mixed anxiety-depressive disorder, 20% substance use disorder, 15% anxiety and/or somatoform disorder, 15% mixed substance related disorder, anxiety and/or depressive disorder, and 10% had major depressive disorder. The Mini-International Neuropsychiatric Interview (MINI) was used to establish Axis I diagnoses [11].

### Instrument

The Structured Clinical Interview for DSM-IV Axis II Personality Disorders (SCID-II) involves a semi-structured interview that assesses ten standard DSM-IV personality disorders, including Depressive PD and Passive-Aggressive PD. The Thai version of SCID-II was developed based on a translation and cultural adaptation process which involved a forward and backward translation carried out by qualified, bilingual staff. The final draft for this study was approved by the author of the original SCID II [4].

### Raters

Nine raters, including 7 psychiatrists, 1 social worker and 1 psychiatry resident made up 8 rater pairs (Table 1). Each subject was randomly selected to be rated by a pair of raters, all of whom were trained in administering the Thai version of SCID II and were supervised by the first and second authors. The training included 2 days of theoretical work, plus an evaluation of video tapes made of 10 subjects not involved in the study. Table 1 shows the 8 pairs of raters that participated in this reliability experiment as well as the number of subjects that each pair rated.

**Table 1:** Pair, rater matches and number of subjects per pair

**Pair Number**	**1**	**2**	**3**	**4**	**5**	**6**	**7**	**8**	**Total**
Rater Names	VU	US	TW	NW	SU	AM	TW	MN	
	MN	SP	SR	SR	TW	SU	VU	TW	
No. of Subjects	19	16	10	8	3	3	2	6	67

### Data analysis

In order to demonstrate the 2 by 2 analysis, only the 4 pairs 1, 2, 3 and 4 were analyzed, while the remaining pairs were not analyzed due to insufficient cell size.

To simplify the formulas used in Cohen’s Kappa and Gwet’s AC1, we created a table showing the distribution of the subjects covered, by rater and response category (Table 2).

**Table 2:** Distribution of subjects - by rater and response category

	**Rater 1**		
**Rater 2**	Category 1	Category 2	Total
Category 1	A	B	B1(A+B)
Category 2	C	D	B2(C+D)
	A1 (A+C)	A2 (B+D)	N

Cohen’s Kappa was calculated using the formula:

$$\frac{p-e\left(K\right)}{1-e\left(K\right)}$$

Where p is the overall percent agreement $\left(p\right)=\frac{A+D}{N}$

*A* = the number of times both raters classify a subject into category 1

*D* = the number of times both raters classify a subject into category 2

*N* = the total sample size

*e(K)*= the chance agreement probability = $\left(\frac{A1}{N}*\frac{B1}{N}\right)+\left(\frac{A2}{N}*\frac{B2}{N}\right)$

Gwet’s AC1 = $\frac{p-e\left(\gamma \right)}{1-e\left(\gamma \right)}$

$$p=\frac{A+D}{N}$$

*e(γ*)= the chance agreement probability = 2q (1-q), $q=\frac{A1+B1}{2N}$

Cohen’s Kappa, Gwet's AC1 and the percentage agreement were calculated using AgreeStat version 2011.3 (Advanced Analytics, Gaithersburg, MD, USA).

## Results

Tables 3 and 4 show the responses of the subjects by rater, response category and percentage of agreement. The overall level of agreement ranged from 84% to 100%, with a mean SD of 96.58 ± 4.99. The most common disagreement among the 4 pairs of raters was in relation to Schizoid and Passive-Aggressive PDs (3 out of the 4 pairs), while the second most common was Dependent, Obsessive-Compulsive and Depressive PDs (2 out of the 4 pairs). None of the PDs showed a 100 percent agreement among the 4 pairs of raters.

**Table 3:** Distribution of subjects by rater and response category for the VU-MN and US-SP pairs of raters

**PDs**		**Rater VU**		**% Agreement**		**Rater US**		**% Agreement**
	**Rater MN**	**No (N)**	**Yes (Y)**		**Rater SP**	**No (N)**	**Yes (Y)**	
Avoidant	No (N)	14	0	100	No (N)	13	0	100
	Yes (Y)	0	5		Yes (Y)	0	3	
Dependent	N	17	1	95	N	15	0	100
	Y	0	1		Y	0	1	
Obsessive-Compulsive	N	12	1	95	N	10	0	88
	Y	0	6		Y	2	4	
Passive-aggressive	N	15	0	100	N	14	0	94
	Y	0	4		Y	1	1	
Depressive	N	15	1	89	N	13	1	94
	Y	1	2		Y	0	2	
Paranoid	N	13	0	100	N	15	0	100
	Y	0	6		Y	0	1	
Schizotypal	N	18	0	100	N	16	0	100
	Y	0	1		Y	0	0	
Schizoid	N	13	2	84	N	15	0	100
	Y	1	3		Y	0	1	
Histrionic	N	17	1	94	N	15	0	100
	Y	0	1		Y	0	1	
Narcissistic	N	19	0	100	N	16	0	100
	Y	0	0		Y	0	0	
Borderline	N	15	0	100	N	14	0	100
	Y	0	4		Y	0	2	
Total Antisocial	N	16	1	89	N	15	0	100
	Y	1	1		Y	0	1	

**Table 4:** Distribution of subjects by rater and response category for the TW-SR and NW-SR

**Pairs of raters**								
**PDs**		**Rater TW**		**% Agreement**		**Rater NW**		**% Agreement**
	**Rater SR**	**No (N)**	**Yes (Y)**		**Rater SR**	**No (N)**	**Yes (Y)**	
Avoidant	**No (N)**	**9**	**0**	**100**	**No (N)**	**7**	**1**	**88**
	**Yes (Y)**	**0**	**1**		**Yes (Y)**	**0**	**0**	
Dependent	N	9	1	90	N	7	0	100
	Y	0	0		Y	0	1	
Obsessive-Compulsive	N	8	0	100	N	6	0	100
	Y	0	2		Y	0	2	
Passive-aggressive	N	9	0	90	N	6	1	88
	Y	1	0		Y	0	1	
Depressive	N	10	0	100	N	7	0	100
	Y	0	0		Y	0	1	
Paranoid	N	9	0	90	N	8	0	100
	Y	1	0		Y	0	0	
Schizotypal	N	9	0	100	N	8	0	100
	Y	0	1		Y	0	0	
Schizoid	N	7	1	90	N	6	0	88
	Y	0	2		Y	1	1	
Histrionic	N	10	0	100	N	8	0	100
	Y	0	0		Y	0	0	
Narcissistic	N	9	0	100	N	8	0	100
	Y	0	1		Y	0	0	
Borderline	N	8	1	90	N	7	0	100
	Y	0	1		Y	0	1	
Total Antisocial	N	10	0	100	N	7	0	100
	Y	0	0		Y	0	1	

Cohen’s Kappa values ranged from 0 to 1.000 (Mean SD = .821 ± .299), whereas Gwet’s AC1 values ranged from .752 to 1.000 (Mean SD = .953 ± .071).

### The effect of trait prevalence

Trait prevalence here was calculated based on the number of positive cases, as judged by both raters, then calculated as a percentage of the total number of cases, and inter-rater reliability (Tables 3, 4 and 5). For example, when calculating the prevalence of Avoidant PD in the VU-MN pair (Table 3), the number of cases in which raters agreed with each other was 5, which was calculated as a percentage of the total number of cases (19), leading to a prevalence rate of 26.32%. Table 6 showed a summary of comparison between Cohen’s Kappa and Gwet’s AC1 values according to prevalence rate for each PD. When the prevalence rate was higher, so were Cohen’s Kappa and the level of agreement; in contrast, the values for Gwet’s AC1 did not change dramatically with prevalence as compared to Cohen’s Kappa, but instead remained close to the percentage of agreement.

**Table 5:** Inter-rater reliability between raters, based on Cohen’s Kappa and Gwet’s AC1

	**VU-MN**		**US – SP**		**TW-SR**		**NW - SR**	
	**Cohen’s**	**Gwet’s**	**Cohen’s**	**Gwet’s**	**Cohen’s**	**Gwet’s**	**Cohen’s**	**Gwet’s**
**PDs**	**Kappa**	**AC1**	**Kappa**	**AC1**	**Kappa**	**AC1**	**Kappa**	**AC1**
Avoidant	1.000	1.000	1.000	1.000	1.000	1.000	0	.858
Dependent	.640	.934	1.000	1.000	0	.890	1.000	1.000
Obsessive-Compulsive	.883	.904	.714	.781	1.000	1.000	1.000	1.000
Passive-Aggressive	1.000	1.000	.636	.924	0	.890	.600	.820
Depressive	.604	.857	.765	.915	1.000	1.000	1.000	1.000
Paranoid	1.000	1.000	1.000	1.000	0	.890	1.000	1.000
Schizotypal	1.000	1.000	1.000	1.000	1.000	1.000	1.000	1.000
Schizoid	.565	.752	1.000	1.000	.737	.840	.600	.820
Histrionic	.641	.938	1.000	1.000	1.000	1.000	1.000	1.000
Narcissistic	1.000	1.000	1.000	1.000	1.000	1.000	1.000	1.000
Borderline	1.000	1.000	1.000	1.000	.615	.866	1.000	1.000
Total Antisocial	.441	.870	1.000	1.000	1.000	1.000	1.000	1.000

**Table 6:** Comparison between Cohen’s Kappa and Gwet’s AC1 according to prevalence rate

**PDs**	**Prevalence rate (%)**	**Cohen’s Kappa**	**Gwet’s AC1**	**% Agreement**
Avoidant	26.32	1.000	1.000	100
	18.75	1.000	1.000	100
	10.00	1.000	1.000	100
	0.0	0.0	.858	88
Dependent	12.50	1.000	1.000	100
	6.25	1.000	1.000	100
	5.26	.640	.934	95
	0.0	0.0	.890	90
Obsessive-Compulsive	31.58	.883	.904	95
	25.00	.714	.781	88
	25.00	1.000	1.000	100
	20.00	1.000	1.000	100
Passive-Aggressive	21.05	1.000	1.000	100
	12.50	.600	.820	88
	6.25	.636	.924	94
	0.0	0.0	.890	90
Depressive	12.50	1.000	1.000	100
	12.50	.765	.915	94
	10.53	.604	.857	89
	0.0	1.000	1.000	100
Paranoid	31.58	1.000	1.000	100
	6.25	1.000	1.000	100
	0.0	1.000	1.000	100
	0.0	0.0	.890	90
Schizotypal	10.00	1.000	1.000	100
	5.26	1.000	1.000	100
	0.0	1.000	1.000	100
	0.0	1.000	1.000	100
Schizoid	20.00	.737	.840	90
	15.79	.565	.752	84
	12.50	.600	.820	88
	6.25	1.000	1.000	100
Histrionic	6.25	1.000	1.000	100
	5.26	.641	.938	94
	0.0	1.000	1.000	100
	0.0	1.000	1.000	100
Narcissistic	10.00	1.000	1.000	100
	0.0	1.000	1.000	100
	0.0	1.000	1.000	100
	0.0	1.000	1.000	100
Borderline	21.05	1.000	1.000	100
	12.50	1.000	1.000	100
	12.50	1.000	1.000	100
	10.00	.615	.866	90
Total Antisocial	12.50	1.000	1.000	100
	6.25	1.000	1.000	100
	5.26	.441	.870	89
	0.0	1.000	1.000	100

For instance, in the VU-MN pair, the prevalence of Depressive PD was 10.53% (2/19 in total), while the Cohen’s Kappa score was .604 (SE .254), Gwet’s AC1 was .857 (SE .104) and the level of agreement was 89%. For the US-SP pair, prevalence was 12.50% (2/16), Cohen’s Kappa was .765 (SE .221) and Gwet’s AC1 was .915 (SE .087), while the level of agreement was 94%.

### Chance agreement probability

The chance agreement probabilities for Cohen’s Kappa (*e(K)*) and Gwet’s AC1 (*e(γ*)) were calculated using the formulae shown above, and in situations where the marginal count was zero (the raters had 100% agreement) as found for the Avoidant, Dependent, Passive-Aggressive and Paranoid PDs in the TW-SR and NW-SR pairs. Cohen’s Kappa gave a ‘0’ value for them all, whereas Gwet’s AC1 gave a value of .858 for Avoidant PD and .890 for the other three PDs – those closest in terms of level of agreement (the Cohen’s Kappa could not be calculated using the SPSS program, due to the fact that at least one variable in each 2-way table upon which measures of association were computed was a constant).

In the first Kappa case, the agreement probability became ‘1’, making the P value equal to ‘0’; whereas, in the case of Gwet’s AC1, the chance agreement probability did not equal ‘0’.

The instance of marginal probability was more apparent for Antisocial and Histrionic PDS within the VU-MN pair. Both pairs had the same prevalence of 5.2% (1/19); however, Antisocial PD had a marginal count of 17 (16+1) for the answer “No,” whilst Histrionic PD had a marginal count of 18 (17+1). Gwet’s AC1 demonstrated higher levels of agreement and higher inter-rater reliability coefficients than Cohen’s Kappa: .870 (SE .095) vs. .441 (SE .330) and with 89% overall agreement for Antisocial PD, and .938 (SE .063) vs. .641 (SE .326) with 94% overall agreement for Histrionic PD. Our analysis documented the robustness of AC1 when used to assess the possibility of marginal problems occurring. Our results confirm those obtained by Gwet [12].

## Discussion

Gwet’s AC1 provides a reasonable chance-corrected agreement coefficient, in line with the percentage level of agreement. Gwet [13] stated that one problem with Cohen’s Kappa is that it gives a very wide range for *e*(*K*) - from 0 to 1 depending on the marginal probability, despite the fact that *e*(*K*) values should not exceed 0.5. Gwet attributed this to the wrong methods being applied when computing the chance agreement probability for Kappa [9].

Clinicians need to be confident that the measures they are using are valid, and poor inter-rater reliability leads to a lack of confidence; for example, in this study Schizoid PD had a high percentage of agreement (88% - 100%) among 4 pairs of raters; therefore, high inter-rater reliability might be expected as well. However, Cohen’s Kappa gave scores of .565, .600, .737 and 1.000, while Gwet’s AC1 gave scores of .757, .840, .820 and 1.000, documenting that a different level of agreement may be reached when these different measures are applied to the same dataset. For example, based on Landis and Koch’s criteria, the Cohen’s Kappa value of .565 falls into the “Moderate” category, while Gwet’s AC1 value of .757 falls into the “Substantial” category (Table 7). A good level of agreement, regardless of the criteria used, is important for clinicians because it supports confidence in the diagnoses being made.

**Table 7:** Benchmark scales for Kappa’s value, as proposed by different investigators

**Landis and Koch**	**Altman**	**Fleiss**
<.0 Poor		
.00 to .20; Slight	<.20 ;Poor	<.40; Poor
.21 to .40; Fair	.21 to .40; Fair	.40 to .75; Intermediate to Good
.41 to .60; Moderate	.41 to .60; Moderate	
.61 to .80; Substantial	.61 to .80; Good	More than .75; Excellent
.81 to 1.00; Almost Perfect	.81 to 1.00; Very Good	

When there are unavoidably low prevalence rates for some of the criteria - a situation which brings about paradox Kappa - it has been found that the number in some cells in the 2×2 table will be small. As shown by Day and Schriger [14], small numbers deviate more from the percentage agreement regression line, while higher numbers deviate less. This is why some researchers use at least 5 cases per cell for their analyses – leaving some criteria with a low prevalence despite the fact that both raters have a high level of agreement [4,6,15-17]. In such cases, some investigators have reported good percentage agreement accompanied by an undesirable Cohen’s Kappa [14]; however, this situation does not occur when using Gwet’s AC1.

It is interesting to note that although Gwet proved that the AC1 is better than Cohen’s Kappa in 2001, a finding subsequently confirmed by biostatisticians [18], few researchers have used AC1 as a statistical tool, or are even aware of it, especially in the medical field. Most recently published articles that have assessed inter-rater reliability have used Cohen’s Kappa exclusively [19-26], and a recent review of the current methods used for inter-rater reliability does not even mention AC1 [27]. During our research of PubMed (up to February 2013), we found only 2 published articles that mention using Gwet’s AC1 method as part of a study [28,29].

Based on the strong evidence shown here of the benefits of using Gwet’s AC1, researchers should be encouraged to consider this method for any inter-rater reliability analyses they wish to carry out, or at least to use it alongside Cohen’s Kappa.

## Conclusions

When assessing the inter-rater reliability coefficient for personality disorders, Gwet’s AC1 is superior to Cohen’s Kappa. Our results favored Gwet’s method over Cohen’s Kappa with regard to prevalence or marginal probability problem.

## Competing interests

The authors declare that they have no competing interest.

## Authors’ contributions

NW and TW conceived of and designed the research. NW supervised the data collection and wrote the manuscript, while DW and KG assisted with the writing of the manuscript. TW and KG were responsible for the statistical analysis. All authors have read and approved the final version of this manuscript.

## Pre-publication history

The pre-publication history for this paper can be accessed here:

http://www.biomedcentral.com/1471-2288/13/61/prepub
